# Higher anxiety and perceived trauma among COVID-19 patients: a prospective comparative study

**DOI:** 10.1186/s12888-023-04574-6

**Published:** 2023-02-09

**Authors:** Alireza Kordi, Atiyeh Sarabi-Jamab, Seyed Vahid Shariat, Nastaran Rezaee, Behnam Shariati, Seyed Hamid Reza Faiz, Fatemeh Sadat Mirfazeli

**Affiliations:** 1 School of Medicine, Iran University of Medical Science, Tehran, Iran; 2 Department of Psychiatry, School of Medicine, Iran University of Medical Sciences, Tehran, Iran; 3 Mental Health Research Center,Psychosocial Health Research Institute, Department of psychiatry, School of Medicine, Iran University of Medical Sciences, Tehran, Iran; 4 Department of Veterinary, Science and Research Branch, Islamic Azad University, Tehran, Iran; 5 Minimally Invasive Surgery Research Center, Department of Anesthesiology and Pain Medicine, School of Medicine, Iran University of Medical Sciences, Tehran, Iran

**Keywords:** COVID-19, Coronavirus, Anxiety, Depression, Perceived trauma, OCD, Obsessions-compulsion-disorder

## Abstract

**Background and purpose:**

Psychiatric disorders such as anxiety, depression, and traumatic stress are not rare during infectious outbreaks, as the COVID-19 pandemic has posed a great concern to the general population. In this study, we aimed to investigate whether experiencing psychiatric symptoms during COVID-19 is the result of the burden of carrying an illness or the COVID-19 itself.

**Method:**

Two hundred ten subjects and three different groups of participants (COVID-19 patients, university staff, and orthopedic patients) were recruited. They answered a demographic questionnaire, Yale-Brown Obsessive–Compulsive Scale (YBOCS) test for OCD symptoms, Impact of Event Scale-Revised (IES-R) for perceived trauma, Beck Anxiety Inventory (BAI) for anxiety, and Beck Depression Inventory (BDI) for depression assessments using phone or face-to-face interviews.

**Result:**

At least one OCD symptom was observed in 85.7% of the subjects. However, there was no significant difference between the 3 groups (*p* = 0.2194). Perceived trauma was significantly higher among COVID-19 patients followed by university staff and orthopedic patients (23.73, 16.21, 11.51 mean IES-R scores respectively, *p* = 8.449e^−14^). COVID-19 patients also showed higher anxiety (mean BAI score: 17.00) than the university staff and orthopedic patients’ group (9.22 and 5.56 respectively) (*p* = 6.175e^−08^). BDI score did not show much variation for depression, the mean score was 9.66, 9.49, and 6.7 for the COVID-19 patients, university staff, and orthopedic patients respectively, (*p* = 0.2735).

**Conclusion:**

Perceived trauma and anxiety symptoms are significantly higher in COVID-19 patients and the symptoms of OCD and depression do not differ between COVID-19 and non-COVID-19 people, so the necessity of screening and following treatment of patients with COVID-19 should be kept in mind.

**Trial registration:**

IR.IUMS.FMD.REC.1399.761.

## Background

The respiratory system is the primary site of infection by the COVID-19 virus. However, many studies have shown that CNS(central nervous system) involvement is a common feature in affected cases of COVID infection. Coronavirus attacks CNS and causes a variety of neuropsychiatric manifestations. This virus in its nature is neurotropic, neuro-invasive, and neurovirulent and it can enter CNS through hematogenous or direct neuron pathways [1]. The olfactory bulb is the most predisposed site of CNS infection as it has a common axonal connection to the nasopharynx and nasal cribriform plate, so the orbitofrontal cortex is affected and because of the constitutional inflammation, the blood barrier’s function will be damaged [1]. physiological factors such as interleukin-1β and medical conditions have been reported to cause neuropsychiatric problems as well [2]. Studies have shown that more than one-third of infected people in the acute phase developed neurologic symptoms and 34% of them showed brain abnormalities such as microhemorrhages, hypodensities, hyperintensities, and infarcts [2].

Remembering the experiences of previous coronavirus pandemics rings the warning bell for the need to pay attention to neuropsychiatric disorders. From 2002-to 2003, the SARS outbreak variety of mental problems including OCD, post-traumatic stress disorder (PTSD), depression, anxiety, somatization, and hostility were reported to increase among other disorders [3, 4]. However, there are no clear and reliable statistics about the influence of Covid-19 and its preventive or curative measures on the general population, but what emerged from reviews describe psychological distresses, sleep disturbances, and affected well-being to a great extent. Goerge Salanti et al. also claim that depression and anxiety are two main domains of mental disorders during the Covid-19 pandemic, have a linear association with the number of SARS CoV-2 infection rates and the stringency of Quarantine, with no regard to age, gender, and country. They also emphasized that the response to the psychological stress generated by the pandemic varied across different populations as they experienced different severity following controlling measures. [5].

Health care protocols such as lockdown, isolation, and emotional distresses have posed a great concern to the mental health of different populations. Therefore, it is understandable that PTSD or traumatic-related stress may occur during the pandemic as we have seen the same circumstances in other disasters [3, 6, 7]. Previous medical conditions predispose patients to experience a more severe form of the disease and in some cases even predict intensive care unit admission so the risks of mental disorders grow to higher levels [8]. Somatic illnesses can increase depression, especially in predisposed subjects; and the severity of this disorder is associated with the severity of the primary disease. In this way, diseases like cancer, coronary syndromes, and stroke, tend to cause a more severe form of depression. This may result in a lower quality of life, social dysfunction, and higher levels of mortality. Appropriate and timely interventions can be helpful and decrease the severity of the problem [9].

Anxiety as the anticipated sequel of illnesses, can be the result of direct effects of the disease on the body or it can be the mental response to the illness, other explanations are that this disorder is the side effect of treatment or it happened by chance [10]. Anxiety not only affects the patients in their acute phase of the illness but also can cause prolonged sequels as we have seen from our previous study that 34.58% of patients presented with anxiety even 9 months after the recovery [7].

However, it is not yet clear whether psychiatric presentation during COVID-19 is a consequence of experiencing a potentially life-threatening disease or the result of the neuro-invasion nature of COVID-19 [11], or what the general population experience due to isolation and all the trauma that happened during COVID-19 pandemic [12].Therefore, in this study, we aimed to compare depression, anxiety, perceived trauma, and obsession in three groups of COVID-19 patients, healthy control, and patients with orthopedic complications. We tried to eliminate the trace of hospitalization on the outcome by having a control group with lower connections to distracting factors on normal brain functions, so we chose orthopedic patients as they have minimal obvious neurological impairments affecting the brain and its functions with a mass burden itself [13].

## Method

### Design

To assess the different impact of COVID-19 on three different populations of COVID-19 patients, university staff and orthopedic patients, we designed this cross-sectional study. All of the subjects were asked to complete a questionnaire either by in-person interview or through an online-based link. A self-administered online questionnaire including the following parts was sent through an SMS to the phone number of the participants after taking consent. Informed consent was obtained from all of the participants.

#### Participants

Our sample included 210 subjects in three distinct groups, 70 COVID-19-infected patients,70 university staff, and, 70 orthopedic non-COVID-19 patients.

COVID-19 patients were chosen out of the registered database for COVID-19-positive patients from three hospitals in Tehran, Iran, through convenient sampling. The inclusion Criterion for this group was being at their acute phase of the disease (between 5 to 21 days of symptoms occurrence) with no history of prior COVID-19 infection. Exclusion criteria were receiving opioid medications, having previous psychiatric disorders resulting in an admission to a psychiatric center, people with severe forms of disease that underwent intubation and experienced mechanical ventilation, and cases with an altered mental status that could not complete the questionnaire.

The orthopedic patients' group who were recruited from orthopedic, non-COVID-19 wards of the same hospitals via convenient sampling. Inclusion criteria were a negative chest CT scan or PCR for COVID-19, being a candidate for an orthopedic procedure, and having no history of COVID-19 or other respiratory infections. Subjects with a history of a psychiatric disorder with admission to a psychiatric center, opioid users, and those with altered mental capabilities that cause an inability to complete the questionnaire were removed from the study.

The university staff group was chosen via convenient sampling from the working staff of the Iran University of Medical Sciences in Tehran. The exclusion criteria for this group were the same as the orthopedic patients' group. However, a PCR test was not taken unless they reported COVID-19 symptoms.

### Assessments

#### Demographic data

Demographic questions included age, gender, education, previous medical conditions such as diabetes mellitus, hypertension, body weight, medications, history of smoking, and use of alcohol, substance, and other medications.

#### OCD symptoms

We used Yale-Brown Obsessive–Compulsive Scale (Y-BOCS) to assess OCD symptoms, which measures 12 different OCD subtypes from aggression-based OCD symptoms to pollution, cleaning, sexual, religious, and other symptoms. This test has acceptable psychometric properties (Cronbach's α of 0.96 and a high-reliability ratio with Pearson's r = 0.94) [14].

#### Perceived trauma

To assess the perceived trauma of COVID-19, we used The Impact of Event Scale-Revised (IES-R). It examines avoidance, hyperactivity, and unwanted thoughts as the three main domains of PTSD. The scale has acceptable reliability and validity. According to a study in Iran, Cronbach's alpha was ranging between 0.84 to 0.93 for each of the three domains [15]. IER-R has 15 items that are scored from 0 to 4 (0 = never, 1 = sometimes, 2 = often, 3 = usually, 4 = always). Higher scores indicate more perceived trauma. Scores more than 23 (24 or more) are considered clinical concerns for PTSD, 33 or more is the cut-off for PTSD diagnosis, and scores more than 36 indicate the most severe degree of perceived trauma with long-lasting effects which may predispose the patients with immune suppression sequels [16].

#### Anxiety symptoms

We used the Beck Anxiety Index (BAI) For the assessment of the severity of anxiety symptoms. It consists of 21 items that each is scored from 0 to 3 (0 = no, 1 = mild, 2 = moderate, 3 = severe). The total score of BAI ranges from 0 to 63; scores from 0 to 7 are considered as negative to minimal symptoms, 8 to 15 scores show a mild form of the disorder, and scores from 16 to 25 and 26 to 63 show moderate and severe levels of anxiety respectively. This test has been validated in Iran and has a Cronbach's alpha of 0.94 and an adequate test–retest reliability over 11 days (*r* = 0.67) [17].

#### Depressive symptoms

We used Beck Depression Index (BDI) for depressive symptoms. This also consists of 21 items that are scored from 0 to 3, and a total score of 0 to 63. A total score of 0 to 13 is considered as the negative or minimal symptoms, 14 to 19 as mild, 20 to 28 as moderate, and 29 to 63 as severe. Ghassemzadeh et al. have validated the test in Iran with acceptable internal consistency (Cronbach's alpha = 0.87) and test–retest reliability (r = 0.74) [18].

#### Statistical analysis

Data were analyzed using SPSS IBM statics version 26 software. We first used descriptive analysis and then a Chi-squared test for the categorical variables of OCD symptoms, and Kruskal–Wallis test by ranks for scaled variables of Depression and anxiety disorders and perceived trauma. To compare each group of non-COVID-19 participants to COVID-19, a T-test, or Wilcoxon rank-sum test was used, when appropriate. A *p*-value < 0.05 was accepted as statistically significant. Moreover, we tried to eliminate the disparity of age, gender and comorbidities effects on the outcomes and compared them between the three groups.

## Results

### Characteristics of the participants

The mean age of subjects was 43.15 ± 15.611 which was higher in the COVID-19 patients’ group (51.86 ± 14.907). Most of the subjects in the COVID-19 patients’ group were in the range of 50 to 69 years of age but the other two groups were younger in the range of 31 to 49 years old.

The frequency of males and females was almost equal in the sample (50.5% and 49.5% respectively) but men were more prevalent in the orthopedic group (62.9%) and females in the university staff group (61.4%). In contrast to the university staff, most of the orthopedic patients were subjects with less educational levels.

Regarding the comorbidity profile, 11.42% of the subjects (24 people) had hypertension and most of them (62.5%) belonged to the COVID-19 group. Similarly, 71.42% of subjects with cardiovascular diseases and 57.89% of subjects with diabetes mellitus were in the COVID-19 group.

History of smoking, alcohol consumption, and opium use was more prevalent in the orthopedic group with 31.4%, 14.3%, and 10% out of 70 subjects respectively.

The demographic status and comorbidity profile of subjects are listed in Table 1.

**Table 1:** Demographic characteristics and comorbidity profile of the participants in the study

		Total	COVID-19 patients	University staff	Orthopedic patients
		*N* (%)	*N* (%)	*N* (%)	*N* (%)
Sex	Male	106 (50.5)	35 (50)	27 (38.6)	44 (62.9)
	Female	104 (49.5)	35 (50)	43 (61.4)	26 (37.1)
Age groups	0–18	6 (2.85)	0 (0)	0 (0)	6 (8.57)
	19–30	45 (21.42)	8 (11.42)	24 (34.28)	13 (18.57)
	31–49	89 (40.95)	23 (32.85)	41 (58.57)	25 (35.71)
	50–69	52 (24.76)	27 (38.57)	5 (7.14)	20 (28.57)
	70 +	18 (8.57)	12 (17.14)	0 (0)	6 (8.57)
Education	unknown	2 (0.95)	2 (2.85)	0 (0)	0 (0)
	Uneducated	67 (31.9)	25 (35.71)	0 (0)	42 (60)
	Diploma	49 (23.3)	20 (28.57)	10 (14.3)	19 (27.1)
	BSC	46 (21.9)	19 (27.14)	18 (25.7)	9 (12.9)
	MSC +	46 (21.9)	4 (5.71)	42 (60)	0 (0)
Co-morbidity	CVD	14 (6.66)	10 (14.28)	1 (1.4)	3 (4.3)
	HTN	24 (11.42)	15 (21.42)	2 (2.8)	7 (10)
	DM	19 (9.04)	11 (15.71)	1 (1.4)	7 (10)
	Asthma	2 (0.95)	2 (2.85)	0 (0)	0 (0)
	Cancer	1 (0.47)	0 (0)	0 (0)	1 (1.42)
	Renovascular	1 (0.47)	1 (1.42)	0 (0)	0 (0)
	Depression	2 (0.95)	1 (1.42)	0 (0)	1 (1.42)
	OCD	1 (0.47)	0 (0)	1 (1.4)	0 (0)
Smoke	Yes	27 (12.9)	4 (5.71)	1 (1.4)	22 (31.4)
	No	183 (87.1)	66 (94.29)	69 (98.6)	48 (68.6)
Alcohol	Yes	12 (5.7)	1 (1.42)	1 (1.42)	10 (14.3)
	No	198 (94.3)	69 (98.58)	69 (98.58)	60 (85.7)
Substance Hx	Yes	7 (3.3)	0 (0)	0 (0)	7 (10)
	No	203 (96.7)	70 (100)	70 (100)	63 (90)

### OCD symptoms

85.7% of subjects showed at least one OCD-related symptom. This was 90.00% in orthopedic patients and 80% in COVID-19 patients, the most prevalent category was pollution with 67.1% of all subjects experiencing it. The descriptive analysis of the OCD symptoms among subjects is listed in Table 2.

**Table 2:** Descriptive analysis of OCD symptoms in three groups of COVID-19 patients ,orthopedic patients and university staff

		Total	COVID-19 patients	University staff	Orthopedic patients
		*N* (%)	*N* (%)	*N* (%)	*N* (%)
OCD	Overall	180 (85.70)	56 (80)	61 (87.1)	63 (90)
	Aggression	95 (45.2)	23 (32.9)	35 (50)	37 (52.9)
	Pollution	141 (67.1)	46 (65.7)	48 (68.4)	47 (67.1)
	Sexual	22 (10.5)	16 (22.29)	3 (4.3)	3 (4.3)
	Store	36 (17.1)	14 (20)	5 (7.1)	17 (24.3)
	Religious	61 (29)	15 (21.4)	24 (34.3)	22 (31.4)
	Symmetry	28 (13.3)	6 (8.6)	11 (15.7)	11 (15.7)
	Miscellaneous	114 (54.3)	32 (45.7)	41 (58.6)	41 (58.6)
	Body	80 (38.1)	29 (41.4)	26 (37.1)	25 (35.7)
	Cleaning	49 (23.3)	17 (24.3)	13 (18.6)	19 (27.1)
	Checking	82 (39)	27 (38.6)	27 (38.6)	28 (40)
	Repeating	34 (16.2)	10 (14.3)	13 (18.6)	11 (15.7)

According to the chi-squared test performed on OCD symptoms of the three groups, no significant difference was observed between the three groups and they showed a similar rate of obsessional symptoms (X^2^ (2, *N* = 70) = 3.0333, *p* = 0.2194). Even a comparison of the COVID-19 patient group to either university staff and orthopedic patients group separately did not unveil any significance with (X^2^ (1, *N* = 70) = 1.3006, *p* = 0.2541)

### Perceived trauma

**Fig. 1:**
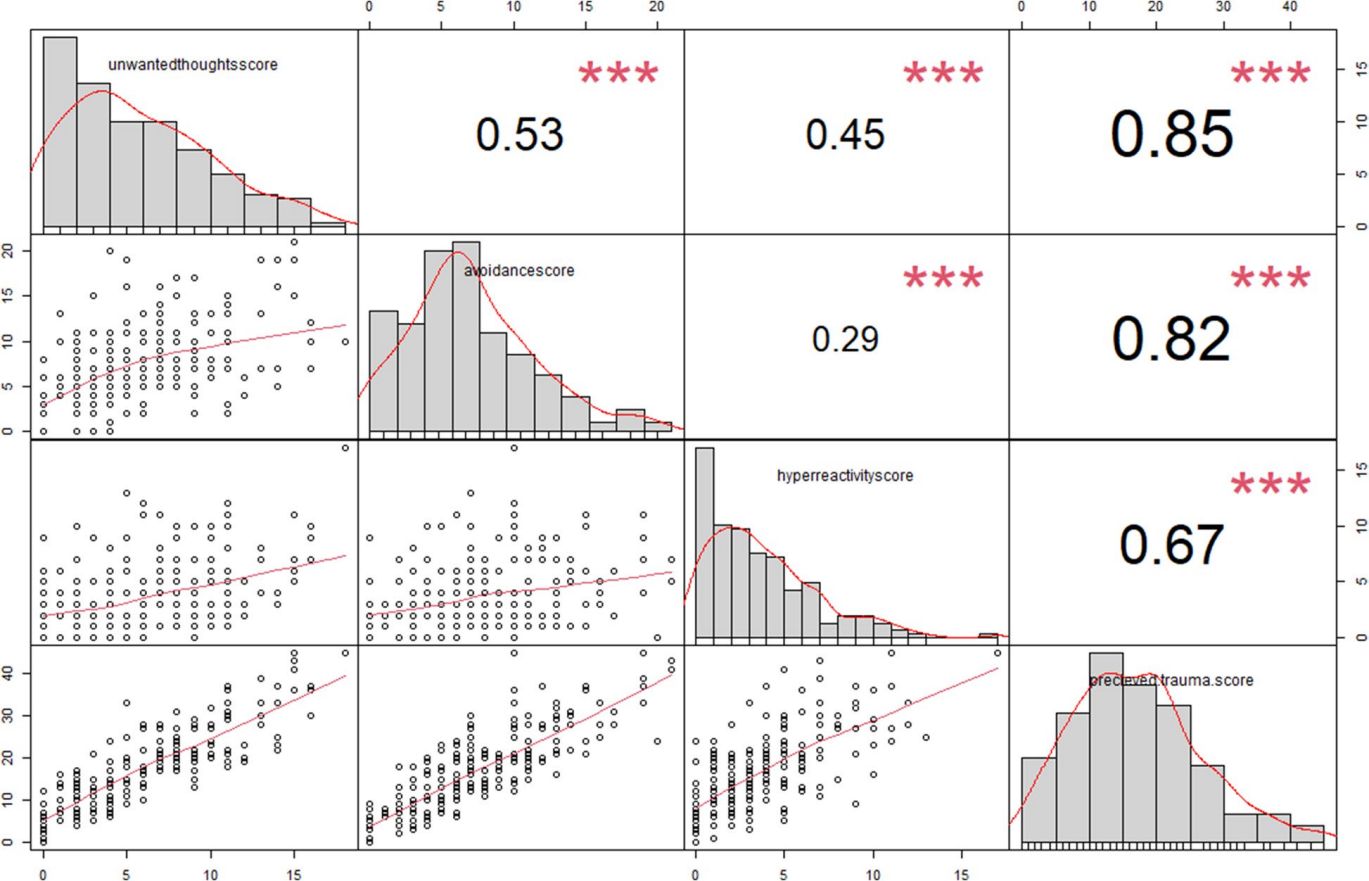
Correlation matrix for overall perceived trauma score. In this figure, the distribution of each variable is shown on the diagonal. On the bottom of the diagonal the bivariate scatter plots with a fitted line are displayed and on the top of the diagonal, the value of the correlation plus the significance level as stars. Each significance level is associated with a symbol as follows: *p*-values of 0, 0.001, 0.01, 0.05, and 0.1, stand for symbols of “***”, “**”, “*”, respectively

We assessed the overall Perceived trauma based on the three main domains of PTSD including avoidance, hyperactivity, and unwanted thoughts. The coefficient correlation for the overall perceived trauma score and its composite scores are shown in the Fig. 1.

Overall Perceived trauma score was 17.15 which was higher in COVID-19 patients (mean = 23.73) than in the other two groups of university staff (mean = 16.21) and Orthopedic patients (mean = 11.51) and As the Kruskal Wallis rank test was performed, COVID-19 group was counted statistically significant in PTSD susceptibility (Chi-square = 60.204, p = 8.449e^−14^, df = 2) (Table 3).

**Table 3:** Descriptive analysis of perceived trauma according to subsets of the disorders

	total	COVID-19 patients	University staff	Orthopedic patients
	Mean (SD)	Mean (SD)	Mean (SD)	Mean (SD)
Unwanted-thought	5.83 (4.248)	8.77 (4.511)	5.44 (3.433)	3.29 (2.649)
Avoidance	7.46 (4.556)	9.56 (5.163)	6.77 (4.202)	6.06 (3.409)
Hyperactivity	3.86 (3.087)	5.40 (3.474)	4.00 (2.283)	2.17 (1.873)
Overall	17.15 (9.426)	23.73 (9.314)	16.21 (8.262)	11.51 (6.090)

78.1% of the subjects presented with less probability of PTSD; while 7.14% passed the PTSD cutoff, 3.8% of them were at higher risk of developing immune dysfunction in the next 10 years. Most of the more severe PTSD survivors belonged to the covid patients’ group (80%), and none of the orthopedic patients reached this cutoff (Table 4).

**Table 4:** Descriptive analysis of perceived trauma in three groups of COVID-19 patients , orthopedic patients and university staff

		total	COVID-19 patients	University staff	Orthopedic patients
		*N* (%)	*N* (%)	*N* (%)	*N* (%)
PTSD cutoffs	Less probability	164 (78.1)	38 (54.28)	58 (82.86)	68 (97.14)
	Clinical concern	31 (14.76)	20 (28.58)	9 (12.86)	2 (2.86)
	PTSD	15 (7.14)	12 (17.14)	3 (4.28)	0 (0)
	Immune dysfunction	8 (3.8)	7 (10)	1 (1.42)	0 (0)

**Fig. 2:**
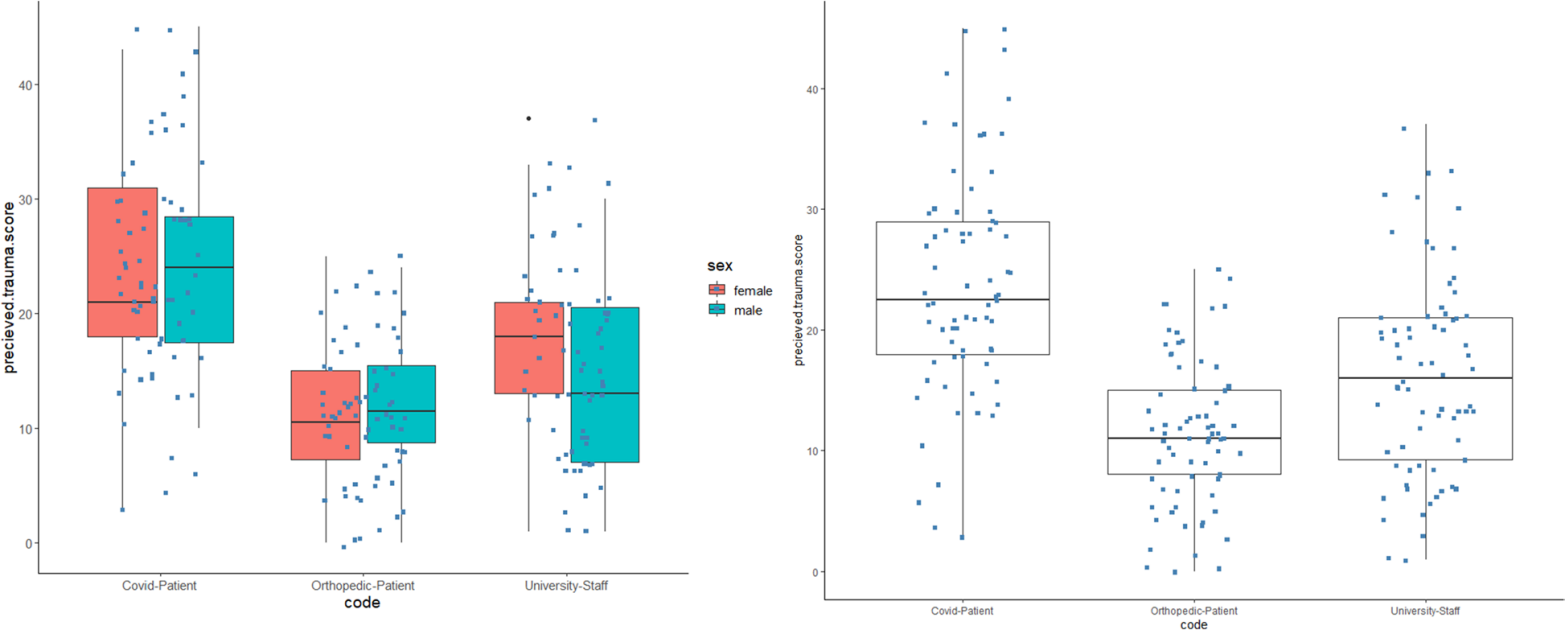
Perceived trauma score scheme and comparison in the study

Then simple T-test was performed to see if COVID-19 patients significantly differ from each of the two other groups; results showed similar outcomes that COVID-19 patients experience more trauma than both university staff and orthopedic patients (t (138) = 5.0496, *p* = *1.376e*^*−*06^) (Fig. 2).

However, through the Table .1, the COVID-19-infected patients have higher proportions of older adults 50 > and a higher number of comorbidities. To control the effect of age, gender, and the number of comorbidities, we used the linear regression on residuals from our model, when they are the bit left over after we subtracted out the predicted values of these predictors. We found a significant difference for different groups; F (2, 207) = 33.06, *p* = 3.4e-13. Thus, the final results were the same as what was previously observed as COVID-19 patients experienced higher trauma than the other groups.

### Anxiety disorder

**Fig. 3:**
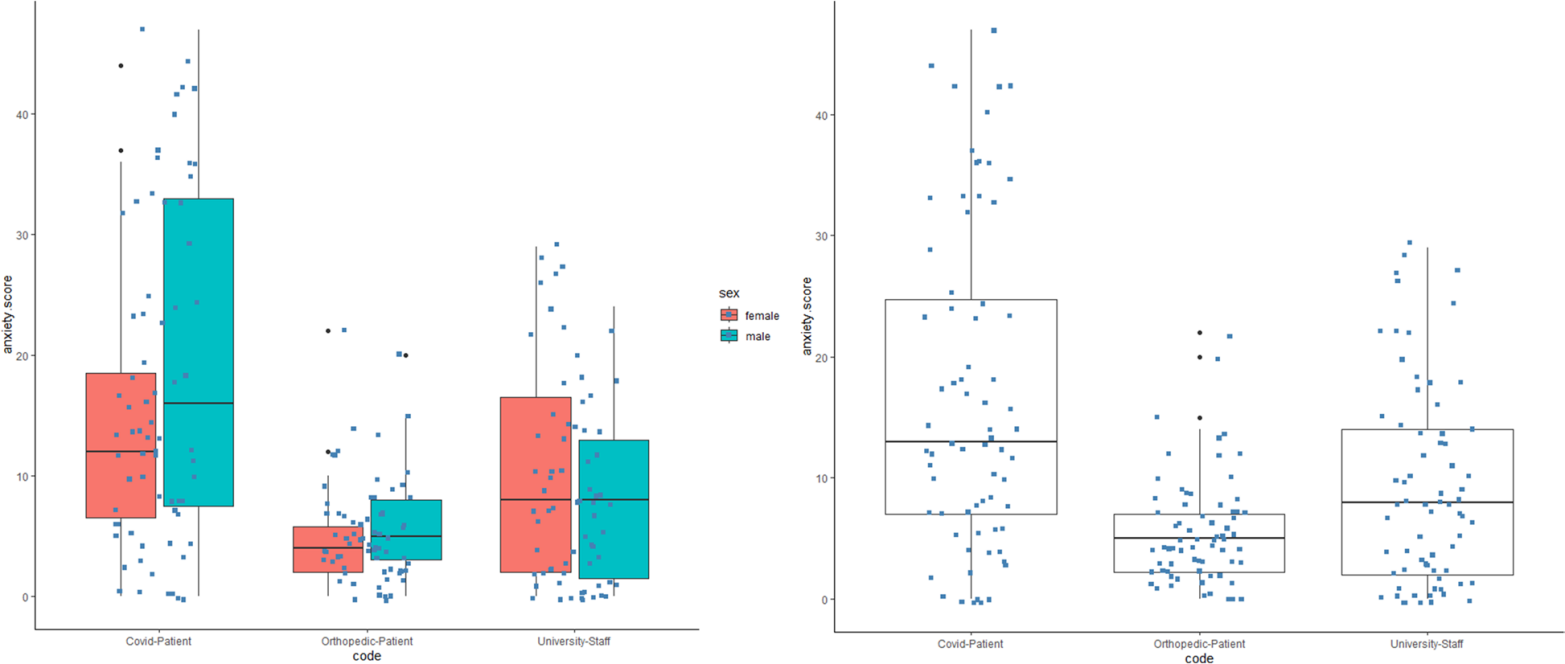
Anxiety score scheme and comparison between three groups of COVID-19 patients, orthopedic patients and university staff in the study

The total mean score of anxiety was 10.59 ± 10.493 that was significantly different between the 3 groups (Chi-square = 33.2, *p* = 6.175e^−08^, df = 2). COVID-19 patients presented with much more anxiety (mean = 17, SD = 13.26) than university staff (mean = 9.2, SD = 8.28) and orthopedic patients (mean = 5.56, SD = 4.44) (*p< *0.001 for both studies) (Fig. 3).

Most of the cases of the COVID-19 patients' group showed moderate anxiety, whereas orthopedic patients demonstrated no to minimal anxiety. Around 50.9% of participants expressed no/minimal symptoms of anxiety, most of them belonged to the orthopedic patients’ group. 77.27% of cases with severe anxiety disorder emerged from the COVID-19 patients’ group (Table 5).

**Table 5:** Descriptive analysis of anxiety disorders in three groups of COVID-19 patients, orthopedic patients and university staff

		total	COVID-19 patients	University staff	Orthopedic patients
		*N* (%)	*N* (%)	*N* (%)	*N* (%)
Anxiety score	Minimal-no symptoms	107 (50.9)	20 (28.57)	34 (48.6)	53 (75.71)
	Mild	55 (26.2)	19 (27.14)	21 (30)	15 (21.43)
	Moderate	26 (12.4)	14 (20)	10 (14.3)	2 (2.86)
	Severe	22 (10.5)	17 (24.29)	5 (7.1)	0 (0)

We again control the effect of age, gender, and the number of comorbidities. We used the linear regression on residuals from our model, and the analysis showed a significant difference for different groups; F(2, 207) = 23, p = 9.5e-10. Thus covid patients still experienced higher anxiety than the two other groups.

### Depressive symptoms

The mean depression score in all of the samples was 8.63 ± 8.107, and a Kruskal Wallis rank-sum test did not show any difference between the three groups (chi-squared 2.593, df = 2, *P*-value: 0.2735) (Table 6).

**Table 6:** Descriptive analysis of depression disorders score in three groups of COVID-19 patients, orthopedic patients and university staff

Depression score	total	COVID-19 patients	University staff	Orthopedic patients
Mean Score (SD)	8.63 (8.107)	9.66 (9.242)	9.49 (8.689)	6.7 (5.722)

**Fig. 4:**
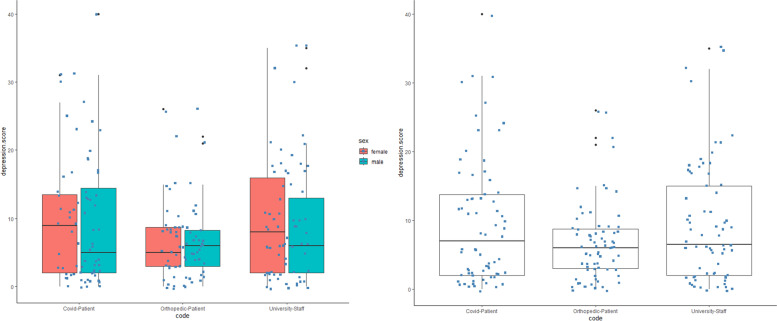
Depression score scheme and comparison in the study

Most of the participants(78.1%) showed no to minimal symptoms of depression with nearly similar numbers to 3 groups. 50% of cases with mild depression symptoms were found in the university staff group. No one from the orthopedic patients’ group declared severe depressive symptoms whereas 5.71% of cases from both the COVID-19 patient group and university staff, had a severe form of this disorder (Table 7, Fig. 4).

**Table 7:** Descriptive analysis of depression disorders in three groups of COVID-19 patients, orthopedic patients and university staff

		total	COVID-19 patients	University staff	Orthopedic patients
		*N* (%)	*N* (%)	*N* (%)	*N* (%)
Depression Score	Minimal-no	164 (78.1)	52 (74.28)	50 (71.42)	62 (88.58)
	Mild	24 (11.4)	8 (11.43)	12 (17.14)	4 (5.71)
	Moderate	14 (6.7)	6 (8.57)	4 (5.72)	4 (5.71)
	Severe	8 (3.8)	4 (5.72)	4 (5.72)	0 (0)

Moreover, controlling the effect of age, gender, and the number of comorbidities through the linear regression on residuals from our model showed that there are no differences between these three groups; F(2, 207) = 23, *p* = 0.7.

## Discussion

This study compared the severity of mental health conditions such as depression, anxiety, perceived trauma, and obsession among three groups of COVID-19, orthopedic patients and healthy subjects during the 4^th^ and 5^th^ coronavirus peaks in Iran to find out whether COVID-19 patients are at higher risk of mental health disorders than other medical conditions and healthy subjects.

COVID-19 patients showed a much higher level of anxiety and perceived stress compared to university staff and orthopedic patients. However, the university staff group reported a higher level of trauma and anxiety respected to orthopedic patients, level of depression and obsession were similar among these three groups. Most of the participants of the orthopedic group were those with lower educational and socio-economic levels as 60% of them were uneducated.University staff (healthy subjects) consisted of younger subjects than the two other groups and the COVID-19 patients’ group had higher co-morbidities than the two other collections. Few studies have reported the effect of educational levels and socio-economic properties on the coping styles with the disease as Lee et al. expressed that depression has an inverse relation to the literacy rate [19]. E. Gonzalo pointed at celibacy and unemployment as the risk factors for higher mental health morbidity besides the female gender and younger ages [20]. However, in our study gender and age did not have any effect on the outcome. informational bias could influence the outcome of the university staff group as some of them were in very close connection with healthcare workers and may have direct access to uncensored news and data about the burden of the disease.

A higher level of anxiety in COVID-19 patients compared to the other two groups, can explain that COVID-19’s anxiety is more than a mere disease's anxiety, and it may suggest involving a neuro-invasion pattern of the virus as previous studies such as one described by Mazza et al., have outlined that according to clinical, laboratory data from cell culture, invitro and those from animal studies, the virus can cause neuroinflammation via direct invasion to CNS or through cytokine storm mechanisms. These findings, besides the concerns of disease confrontations, uncertainty about the future, and traumatic memories from a severe experiment of the illness, can cause disorders ranging from simple mood disorders to anxiety and even psychosis in susceptible subjects. Cytokine disturbances like dysregulation of IL-6, IL-1, IFN-ϒ, and TNF-α have been reported to be influencing these conditions [21].

However higher level of perceived trauma can also explain that COVID-19 is a traumatic disease with an unknown outcome that could impact mental health gravely. Consistent with previous studies, perceived trauma was observed highly in the infected group and most of them showed avoidance presentation. Buthaina Al Falasi et al. did a study on Italian citizens by using Startle physiological arousal and anger numbness and they showed 43.8% of PTSD symptoms (compared to ours, 7.14%). They also endorsed that their estimation of the prevalence of PTSD was higher than an actual percentage as they used a screening tool instead of the diagnostic one. They pointed to female sex and younger ages as the risk factors for developing PTSD [3]. Buthaina Al Falasi in another study mentioned quarantine and suffering a family member or close friend from COVID-19 infection as another precipitating factor in developing PTSD at younger ages [22]. Zhang L and Cénat JM reported a prevalence of 15% to 22% for PTSD after the pandemic [23, 24]. Another study in Italy stated 23.5 percent for the prevalence of PTSD among the general population in the early COVID-19 era [25]. The difference between our findings from other studies could be firstly attributed to the different societies in which the experiments were done, next explanation is that the fear of people from the burden of COVID-19 reduced after 3 to 4 waves of the disease. Mona Salehi et al. reported the prevalence of PTSD as 1.1% for the general population in 2014 in the normal non-pandemic era. They also endorsed the prevalence of definite PTSD as 36%, 18%, and 9% for MERS, SARS, and COVID-19 accordingly. At last, they estimated that near 29% of people who survive from the infection experience levels of PTSD symptoms [26]. In a study close to ours, E. Seyahi et.al in turkey assessed the effect of Covid-19’s burden on patients with rheumatoid diseases versus hospital workers and high school teachers during the first months of the outbreak. They claimed that rheumatoid patients (like school teachers) faced less anxiety, depression and PTSD symptoms compared to hospital workers. In their study, they did not discriminate between COVID and non-COVID subjects as there were 21 subjects with Covid 19 infection distributed in those 3 groups. They emphasized that despite lower levels of psychiatric symptoms in rheumatoid patients against hospital workers, there is a significant concern over the traumatic impact of COVID-19 on those patients as the mean score of IES-R score was higher than expected [27].In another study, F. Ingegnoli et al., stated that Covid-19 with IES-R score of 29.7 was considered responsible for a massive psychosocial burden on rheumatoid patients with a prevalence of 41% PTSD symptoms. They stressed the role of socioeconomic on the subjects and claimed that the higher income is associated with lower levels of stress apprehended after the pandemic [28].

However, depression symptoms were observed similarly between three groups of people, with an overall rate of 21.9%, and most of them experienced mild symptoms. One might say that we assessed patients in the acute phase and it may take time that patients to develop depressive symptoms but stress reaction and anxiety to the disease will develop sooner. We also can correlate this insignificance to the ceiling effect and the nationwide prevalence of depression as the low social support for the whole community has stressed the general population. Another explanation is the low sensitivity of our screening tool to distinguish between acute onset depression and chronic forms of it. A 1-month cohort study done by Mazza et al. demonstrated that COVID-19 patients are at higher risk of developing depressive disorders [21], so the need for a follow-up study is sensed.

Our patients did not show many differences in their obsessional symptoms either, however their range of obsessive symptoms was high. OCD symptoms were observed in 85.7% of all three groups with no superiority for any of them, 80% of COVID-19 patients showed at least one OCD-related symptom with the majority of 67.1 for pollution-related symptoms such as environmental dirt or even body-related discharges. Two other Subgroups of the study showed about the same percentages. Higher rates of symptoms associated with washing are reasonable because such habits are more encouraged by social pressure to limit the transition pathways. Accordingly Adam abba aji et al., did a study to evaluate the prevalence of OCDs in the general population of Canada during the recent outbreak; they conducted an online survey by using a brief obsessive–compulsive scale (BCOS) which showed 60.3% of participants with new presentations of symptoms, 53.8 had compulsion of hand washing. This was beside 25.1 percent of the population with prior symptoms. So, the overall percentage equaled 85.4% which was too similar to our study with 85.7%. The authors declared that the population of their study was more exposed to general anxiety disorders (GAD) and major depressive disorders (MDD) [29]. A review done by Mina Soleimani and her colleague suggests that an increase in cleaning activities and a decrease in physician visits regarding lowering contamination risks puts a higher danger of worsening mental disorders, especially OCD. This article estimates the total definite OCD prevalence as defined by the criteria, is about 2% with variations by ages as young adults experience a higher rate than other group ages. Accordingly GAD and panic attacks were seen more in women with OCD during the corona pandemic. Therefore, fear of contamination, continues and repetitive hand washing, isolation, uncertainty about future, need for doing routine orders of daily tasks that may be not possible during the pandemic, and aggressive thoughts about harming the nearby people could be suggested as the base for incidence of OCD [30].

### Limitation

Cross-sectional studies do not represent cause-effect relations between the variables. The restrictions caused by the pandemic state, then the heterogenicity between the socio-educational level of the groups especially the orthopedic patient group and university staff group may enforce biases on the results. Next, As the first goal of admission of COVID-19 patients is to save their life, our study could not have an estimation of the more severe cases so our study is limited to the non-ill, stable patients.

## Conclusion

Our study in line with the literature, confirms that COVID-19 causes mental disturbances such as anxiety and trauma in the infected people compared to the controlled groups, but it is not yet clear whether an organic pathology is underway for the CNS involvement or these processes are because of the fear and burden of the disease or both. Perceived trauma and anxiety symptoms are significantly higher in COVID-19 patients and the symptoms of OCD and depression do not differ between COVID-19 and non-COVID-19 people. Therefore, all COVID-19 patients should be screened for psychological complications and preventive measures such as a positive atmosphere in the corona ward and psychological counseling for vulnerable patients should be implemented. Treatment of psychologic complications in patients with COVID-19 should be kept in mind.

## Data Availability

The datasets used and analyzed in the current study are available from the corresponding author upon reasonable request.

## Abbreviations

COVID-19: Coronavirus disease of 2019
Y-BOCS: Yale Brown Obsessive-Compulsive Scale
OCD: Obsessive compulsive disorder
IES-R: Impact of Event Scale - Revised
BAI: Beck Anxiety Inventory
BDI: Beck Depression Inventory
CNS: Central Nervous System
SARS: Severe Acute Respiratory Syndrome
PTSD: Post-traumatic stress disorder
